# Single-inhaler triple therapy in patients with chronic obstructive pulmonary disease: a systematic review

**DOI:** 10.1186/s12931-019-1213-9

**Published:** 2019-11-04

**Authors:** Sue Langham, Jen Lewis, Nick Pooley, Nina Embleton, Julia Langham, MeiLan K. Han, James D. Chalmers

**Affiliations:** 1 Maverex Limited, Manchester, UK; 2 University of Michigan, Women’s Respiratory Clinic, Ann Arbor, USA; 3 University of Dundee and Ninewells Hospital and Medical School, Dundee, UK

**Keywords:** Chronic obstructive pulmonary disease, Inhalers, Combination drug therapy, Disease exacerbation

## Abstract

**Background:**

Guidelines recommend that treatment with a long-acting β_2_ agonist (LABA), a long-acting muscarinic antagonist (LAMA), and inhaled corticosteroids (ICS), i.e. triple therapy, is reserved for a select group of symptomatic patients with chronic obstructive pulmonary disease (COPD) who continue to exacerbate despite treatment with dual therapy (LABA/LAMA). A number of single-inhaler triple therapies are now available and important clinical questions remain over their role in the patient pathway. We compared the efficacy and safety of single-inhaler triple therapy to assess the magnitude of benefit and to identify patients with the best risk-benefit profile for treatment. We also evaluated and compared study designs and population characteristics to assess the strength of the evidence base.

**Methods:**

We conducted a systematic search, from inception to December 2018, of randomised controlled trials (RCTs) of single-inhaler triple therapy in patients with COPD. The primary outcome was the annual rate of moderate and severe exacerbations.

**Results:**

We identified 523 records, of which 15 reports/abstracts from six RCTs were included. Triple therapy resulted in the reduction of the annual rate of moderate or severe exacerbations in the range of 15–52% compared with LAMA/LABA, 15–35% compared to LABA/ICS and 20% compared to LAMA. The patient-based number needed to treat for the moderate or severe exacerbation outcome ranged between approximately 25–50 (preventing one patient from having an event) and the event-based number needed to treat of around 3–11 (preventing one event). The absolute benefit appeared to be greater in patients with higher eosinophil counts or historical frequency of exacerbations and ex-smokers. In the largest study, there was a significantly higher incidence of pneumonia in the triple therapy arm. There were important differences in study designs and populations impacting the interpretation of the results and indicating there would be significant heterogeneity in cross-trial comparisons.

**Conclusion:**

The decision to prescribe triple therapy should consider patient phenotype, magnitude of benefit and increased risk of adverse events. Future research on specific patient phenotype thresholds that can support treatment and funding decisions is now required from well-designed, robust, clinical trials.

**Trial registration:**

PROSPERO #CRD42018102125.

## Background

Chronic obstructive pulmonary disease (COPD) is a leading cause of mortality and morbidity worldwide [1]. Pharmacological treatment relies predominately on inhaled bronchodilators and anti-inflammatory agents [2]. The 2019 Global Initiative for Chronic Obstructive Lung Disease (GOLD) strategy document, based on the best-available evidence from the published literature, recommend that choice of treatment depends on symptom and exacerbation severity. In those patients with a high risk of exacerbations, therapy relies on a long-acting muscarinic antagonist (LAMA) or if a patient is highly symptomatic, dual therapy with a LAMA and a long-acting β_2_ agonist (LABA). Evidence suggests that dual therapy with LABA and inhaled corticosteroids (ICS) and the step up to triple therapy (LAMA/LABA/ICS) be considered for a select group of patients who continue to exacerbate despite appropriate treatment and/or features suggesting steroid responsiveness.

Triple therapy, provided as multiple inhalers, has in pooled analyses been shown to improve lung function, health-related quality of life and exacerbations [3–7]. However, evidence suggests that triple therapy is often over prescribed in clinical practice and used in patients who are not frequent exacerbators [8–10]. Recently, two single-inhaler triple therapies have received marketing authorisation from the European Medicines Agency and one other is in late-stage clinical development [11]. A systematic and critical review of the evidence base for single-inhaler triple therapy is warranted to support clinical decision making for the following reasons. First, important questions in clinical practice remain over the role of triple therapy, which include the magnitude of clinical benefit and the identification of patients with the best risk-benefit profile for treatment. Second, the randomised controlled trial data for single-inhaler therapies will be used to inform the evidence base for triple therapy as a whole, therefore it is important to understand its strengths and limitations. To the best of our knowledge there are currently no systematic reviews that give a comprehensive and critical assessment of the evidence base for single-inhaler triple therapy.

Following the GOLD 2019 update, such a review would provide insights into patients most suited to triple therapy, the strength of the evidence base and the need for future research. The main objective of this study was to conduct a systematic review of randomised controlled trials comparing the efficacy and safety of fixed-dose combinations of a LABA, LAMA and ICS (single-inhaler triple therapy) with LABA, LAMA, LABA/LAMA or LABA/ICS for the treatment of adult patients with COPD. We present the relative and absolute benefit of single-inhaler triple therapy overall and for specific patient subgroups for each trial. We also present estimates of number needed to treat (NNT) and an overview of risks associated with treatment to support clinical decision making. In addition, we present and discuss the characteristics of each trial, highlighting how study design features could impact the interpretation of clinical trial results and cross-trial comparisons.

## Methods

We followed the Preferred Reporting Items for Systematic Reviews and Meta Analyses (PRISMA) guidelines for conducting and reporting systematic reviews [12]. The study protocol was prepared and published via PROSPERO (#CRD42018102125) [13]. Eligibility criteria included (see Additional file 1: Table S1): population of adult patients with COPD; intervention of single-inhaler triple therapy; comparators of LABA, LAMA, LAMA/LABA or LABA/ICS; and study design of parallel-group randomised controlled trials of ≥3 months duration. Single-inhaler triple therapies of interest included glycopyrronium bromide/formoterol fumarate/beclomethasone (GLY/FOR/BDP; Trimbow®), umeclidinium/vilanterol/fluticasone furoate (UMEC/VI/FF; Trelegy Ellipta), GLY/FOR/budesonide (GLY/FOR/B; PT010) and GLY/indacaterol/mometasone furoate (GLY/IND/MF; QVM149). The primary outcome of interest was the annual rate of moderate and severe exacerbations. Moderate exacerbations were those that required treatment with systemic corticosteroids and/or antibiotics. Severe exacerbations were those that required hospitalisation or resulted in death [14]. Secondary outcomes included time to first exacerbation, lung function, quality of life and safety outcomes (including serious adverse events [SAEs], pneumonia and mortality).

Searches for full-text reports and conference abstracts containing original data (in English) were run in: MEDLINE, EMBASE, Cochrane Central Register of Controlled Trials, ClinicalTrials.gov and the World Health Organization International Clinical Trial Registry Platform. The final update was in December 2018. The detailed search strategy is available in Additional file 1: Table S2.

Two reviewers (SL and JLe) independently screened the titles and abstracts of citations, and then full-text reports/conference abstracts according to the protocol. Those studies, containing original data, which met the eligibility criteria of this review were included. Disagreements were resolved through consensus or consultation with a third reviewer (JLa or NP). Data were extracted by one author (JLe) and checked independently by two reviewers (SL and NP). Disagreement between the authors was solved by consensus or involvement of a fourth reviewer (JLa). For the primary outcome we extracted data for the overall population and for several phenotypic subgroups including prior exacerbation frequency, blood eosinophil levels/counts and smoking status.

The risk of bias was assessed according to recommendations outlined in the Cochrane Handbook for Systematic Reviews of Interventions, and graded [15]. The tool includes an assessment of sequence generation, concealment of allocation, blinding of participants and investigators, incomplete outcome data and selective outcome reporting. Each source of bias was graded as having high, low or unclear risk.

Data assimilation on the primary and secondary outcomes was through descriptive analysis in the form of tables, figures and descriptive forest plots. Rate ratios (RRs) for the comparison of the annual rate of moderate and severe exacerbations between triple therapy and comparators were displayed, for each study separately, on a forest plot for the overall trial population and for phenotypic subgroups. Where RR and 95% confidence intervals (CIs) were not available from each study they were calculated from the rate of annual moderate or severe exacerbation for triple therapy and comparators and the number of patients per subgroup using the RR in ‘R’ version 3.3.2. Where RR were available from each study, but 95% CIs were graphically displayed, we approximated the CIs from the forest plots reported in the original publication using graphical software (GetData Graph Digitizer).

We also calculated patient- and event-based NNT for the primary outcome for each of the 12-month studies. Patient-based NNT demonstrates the number of patients that need to be treated with triple therapy relative to the comparator to prevent one patient from having ≥1 moderate or severe exacerbation over one year. As the proportion of patients with ≥1 exacerbation was rarely reported, proportions were approximated from the Kaplan–Meier curves for the time to the first exacerbation using graphical software (GetData Graph Digitizer). Event-based NNT demonstrates the number of patients that need to be treated to prevent one moderate or severe exacerbation. These were calculated based on the annual rate of moderate or severe exacerbations.

## Results

**Fig. 1:**
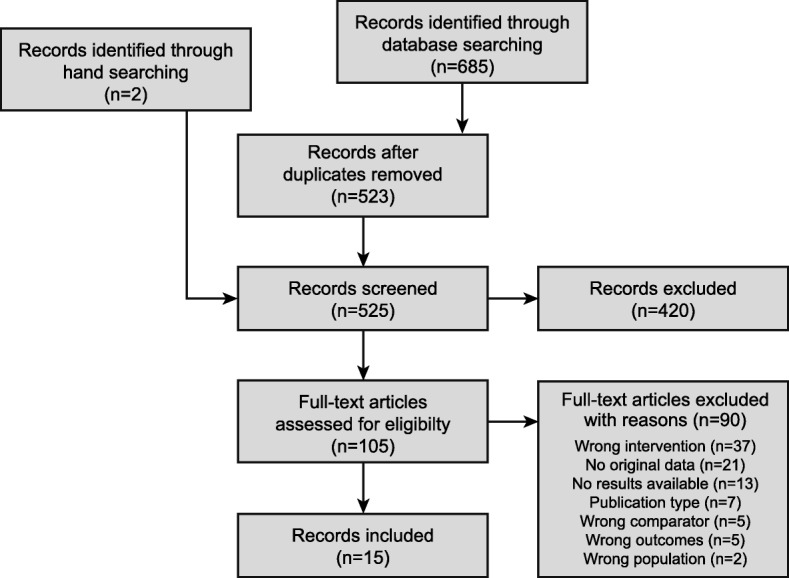
Flow chart of search results

The initial search returned 523 references after de-duplication. From these, we identified 105 as potentially relevant. A full-text analysis led to the removal of 90, leaving 15 records (seven abstracts/posters) belonging to six studies for inclusion (Fig. 1) [16–30]. Detailed reasons for exclusion are outlined in Additional file 1: Table S3. The majority of studies were excluded because they reported results for interventions or comparators not included in the eligibility criteria.

The characteristics of the included studies are displayed in Table 1. The six studies included in this review had enrolled 19,658 participants with COPD (range 1810 to 10,355). All of the included studies were multinational, randomised, double-blind, parallel-group trials. Four of the six studies were 1-year duration (TRINITY [16], TRILOGY [17], TRIBUTE [23] and InforMing the PAthway of COPD Treatment [IMPACT] [25]). The primary endpoint in one study was assessed after 24 weeks, but a subset of patients continued treatment for ≤1 year (Lung FUnction and quality of LiFe assessment in COPD with closed triple therapy [FULFIL] [24]). The final study was 24-weeks duration (KRONOS [26]).

**Table 1:** Overview of included studies and study characteristics at baseline

Study	Treatment	Key comparisons	Study length	Number of patients	FEV_1_, post-bronchodilator, % of predicted normal value	CAT score		Exacerbation rate (range)	Exacerbations in the previous year			Prior triple therapy^c^	Prior ICS	Patients with asthma
						≥10	Mean (SD)		≥1 moderate or severe	≥2 moderate or severe	≥1 severe			
TRINITY [16]	GLY/FOR/BDP (Trimbow)	Triple therapy (single inhaler) versus TIO^a^	52 weeks	2691	36.6%	100%	21.5 (5.8)	1.3 (1–11)	NR	NR	NR	Excluded	77%	Excluded
	TIO				36.6%	100%	21.6 (5.8)	1.3 (1–5)	NR	NR	NR		78%	
	FOR/BDP + TIO				36.7%	100%	21.7 (6.0)	1.2 (1–7)	NR	NR	NR		73%	
TRILOGY [17]	GLY/FOR/BDP (Trimbow)	Triple therapy versus LABA/ICS	52 weeks	1368	36.9%	100%	20.8 (5.9)	1.2 (1–5)	NR	NR	NR	Excluded	75%	Excluded
	FOR/BDP				36.2%	100%	20.8 (5.7)	1.2 (1–6)	NR	NR	NR		73%	
TRIBUTE [23]	GLY/FOR/BDP (Trimbow)	Triple therapy versus LAMA/LABA	52 weeks	1532	36.4%	100%	NR	1.2 (1–6)	100%	20%	NR	Excluded	66%	Excluded
	GLY/IND				36.4%	100%	NR	1.2 (1–4)	100%	18%	NR		64%	
FULFIL^b^ [24]	UMEC/VI/FF (Trelegy Ellipta)	Triple therapy versus LABA/ICS	24 weeks (extension to 52 weeks)	1810 (430)	47.1%	100%	NR	NR	70%	34%	NR	32%	66%	Excluded
	FOR/B + placebo				45.4%	100%	NR	NR	67%	31%	NR	33%	67%	
IMPACT [25, 31]	UMEC/VI/FF (Trelegy Ellipta)	Triple therapy versus LAMA/LABA Triple therapy versus LABA/ICS	52 weeks	10,355	45.7%	100%	20.1 (6.1)	NR	99.95%	55%	26%	38%	72%	Excluded^d^
	UMEC/VI				45.4%	100%	20.2 (6.2)	NR	99.90%	55%	25%	40%	72%	
	VI/FF				45.5%	100%	20.1 (6.1)	NR	99.88%	54%	26%	38%	70%	
KRONOS [26]	GLY/FOR/B	Triple therapy versus LAMA/LABA Triple therapy versus LABA/ICS	24 weeks	1902	NR	100%	18.7 (6.4)	0.4 (0.8)^e^ 0.0 (0–8)^f^	27%	7%	NR	31%	72%	Excluded
	GLY/FOR				NR	100%	18.1 (6.1)	0.3 (0.7)^e^ 0.0 (0–5)^f^	24%	7%	NR	28%	71%	
	FOR/B				NR	100%	18.4 (6.6)	0.3 (0.6)^e^ 0.0 (0–4)^f^	25%	6%	NR	34%	71%	

*B* budesonide, *BDP* beclomethasone, *CAT* COPD assessment test, *COPD* chronic obstructive pulmonary disease. *FEV*_*1*_ forced expiratory volume in one second, *FF* fluticasone furoate, *FOR* formoterol fumarate, *FULFIL* Lung FUnction and quality of LiFe assessment in COPD with closed trIpLe therapy, *GLY* glycopyrronium bromide, *ICS* inhaled corticosteroids, *IMPACT* InforMing the PAthway of COPD Treatment, *IND* indacaterol, *LABA* long-acting β_2_ agonist, *LAMA* long-acting muscarinic antagonist, *NR* not reported, *SD* standard deviation, *TIO* tiotropium, *UMEC* umeclidinium, *VI* vilanterol

^a^Other comparisons were triple therapy (single inhaler) versus LABA/ICS + LAMA and TIO versus LABA/ICS + LAMA

^b^52-week data

^c^Including LAMA, LAMA and ICS

^d^Patients with a prior history of asthma, but not those with a current diagnosis of asthma, were eligible for inclusion

^e^Mean (SD)

^f^Median (range)

The mean age of participants in the different studies was relatively similar (63.3–65.9 years). All the studies had more male than female participants (66–77% males). Where reported, the duration of COPD was similar between five of studies (7.7–8.2 years); the duration of COPD in the KRONOS study was slightly lower (5.4–6.2 years).

Disease-specific study characteristics are outlined in Table 1. Participants’ post-bronchodilator forced expiratory volume in one second (FEV_1_) was 36% of predicted normal value in three of the studies (TRINITY, TRILOGY and TRIBUTE), and 45–47% in two of the studies (FULFIL and IMPACT); the percentage predicted post-bronchodilator FEV_1_ was not reported in the KRONOS study. In terms of symptoms, patients from all the studies were required to have a baseline COPD assessment test (CAT) score of ≥ 10 (CAT scores range from 0 to 40). The baseline CAT score was 20–21 in the TRINITY, TRILOGY, TRIBUTE and IMPACT studies; and 18–19 in the KRONOS study (the FULFIL study did not report a mean baseline CAT score).

Patients from all studies, except the FULFIL and KRONOS studies, were required to have had ≥ 1 exacerbation in the previous year. Patients enrolled into the TRINITY, TRILOGY and TRIBUTE studies all had ≥ 1 moderate or severe exacerbation in the previous 12 months. The FULFIL study inclusion criteria were either FEV_1_ < 50% predicted or ≥ 2 moderate or ≥ 1 severe exacerbation with FEV_1_ 50–80% predicted; and the IMPACT study inclusion criteria were either ≥ 1 moderate or severe exacerbation with FEV_1_ < 50% predicted, or ≥ 2 moderate or ≥ 1 severe exacerbation(s) with FEV_1_ 50–80% predicted. Patients in the KRONOS study were not required to have had a COPD exacerbation within the previous year. Exacerbation rate was a primary objective for the TRINITY, TRIBUTE and IMPACT studies and a secondary objective for the TRILOGY, FULFIL and KRONOS studies (lung function was the primary endpoint for these studies).

Three of the studies (TRINITY, TRILOGY and TRIBUTE) excluded patients who were already on triple therapy and did not permit other COPD treatments during the trial; however, 64–78% of patients had received ICS-containing regimens prior to the study start. The FULFIL and IMPACT studies included patients who were already on triple therapy and allowed patients to take their existing medications during the run-in period. Around 32–40% of patients were receiving triple therapy during the run-in period and 66–72% of patients were receiving a regimen that included an ICS. In the KRONOS study, 23–32% of patients had previously taken triple therapy, and were not permitted to take LAMA, LABA, LAMA/LABA or LABA/ICS during the trial; 71–73% of patients were receiving ICS at screening.

All of the studies excluded patients with a current diagnosis of asthma. Patients with a prior history of asthma were eligible to enrol in the IMPACT study if they had a current diagnosis of COPD.

The studies generally had a low risk of bias according to the Cochrane risk of bias tool [15] (see Additional file 1: Figure S1). There was an unclear risk of bias for selection (allocation concealment), performance bias (blinding of participants and personnel) and detection bias (blinding of outcome assessment) in the FULFIL study due to insufficient information. For the IMPACT study, there was an unclear risk of detection bias (blinding of outcome assessment) also due to insufficient information. The TRINITY, TRILOGY and TRIBUTE studies were funded by Chiesi Farmaceutici, the FULFIL and IMPACT studies were funded by GlaxoSmithKline, and the KRONOS study was funded by Pearl Therapeutics – a member of the AstraZeneca Group.

### Primary outcome

**Fig. 2:**
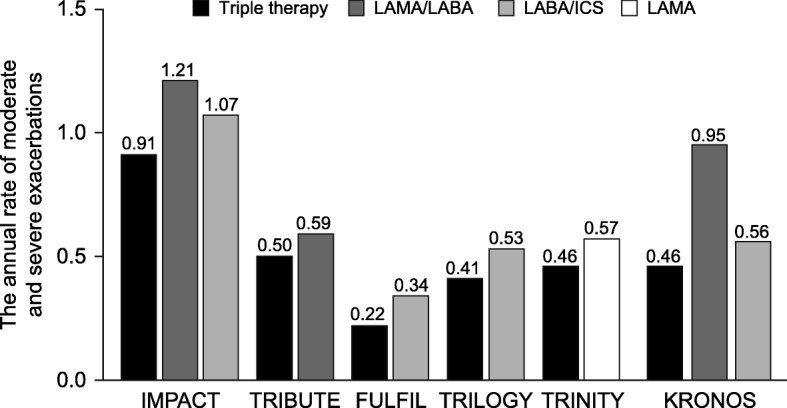
Annual rate of moderate and severe exacerbations. *COPD* chronic obstructive pulmonary disease, *FULFIL* Lung FUnction and quality of LiFe assessment in COPD with closed trIpLe therapy, *ICS* inhaled corticosteroids, *IMPACT* InforMing the PAthway of COPD Treatment, *LABA* long-acting β_2_ agonist, *LAMA* long-acting muscarinic antagonist

**Fig. 3:**
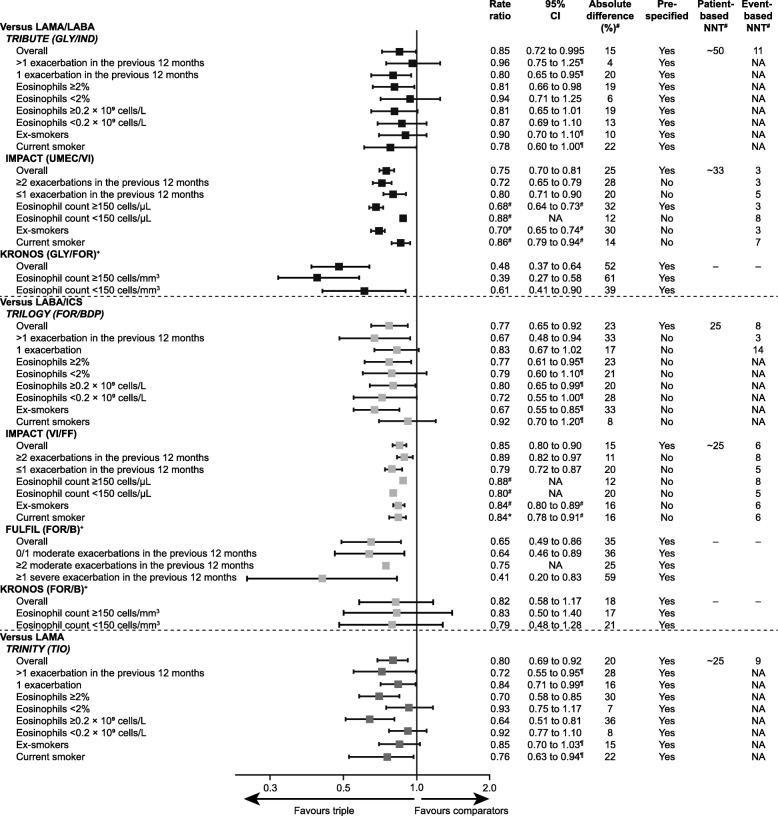
Descriptive presentation of the annual rate of moderate and severe exacerbations for within-trial comparisons. *B* budesonide, *BDP* beclomethasone, *CI* confidence interval, *COPD* chronic obstructive pulmonary disease, *FF* fluticasone furoate, *FOR* formoterol fumarate, *FULFIL* Lung FUnction and quality of LiFe assessment in COPD with closed trIpLe therapy, *GLY* glycopyrronium bromide, *ICS* inhaled corticosteroids, *IMPACT* InforMing the PAthway of COPD Treatment, *IND* indacaterol, *LABA* long-acting β_2_ agonist, *LAMA* long-acting muscarinic antagonist, *NA* not available, *NNT* number needed to treat, *TIO* tiotropium, *UMEC* umeclidinium, *VI* vilanterol. ^#^Calculated. ^¶^Estimated from graph. ^+^A 24-week study. Rate ratios, 95% CIs and NNT for annual rate of moderate and severe exacerbations between triple therapy and comparators, overall and by subgroups for each within-trial comparison of triple therapy and LAMA/LABA, LABA/ICS and LAMA. Cross-trial comparisons are descriptive only. NNT were only calculated for 52-week studies, ~ numbers used in calculation estimated from graph

The rate of moderate or severe exacerbations during treatment among patients assigned to each intervention is outlined in Fig. 2. The rates were much higher in the IMPACT study and the LAMA/LABA group in the KRONOS study compared with the other studies. Figure 3 outlines the RR, 95% CIs and patient- and event-based NNTs for triple therapy compared with each comparator for each study for the overall population and each subgroup for the primary outcome. The graph is a descriptive display of the data for each study.

#### Overall population

For overall populations, the rate of moderate or severe exacerbations was generally significantly lower for triple therapy than the comparators. In the studies that assessed single-inhaler triple therapy with a LAMA/LABA (GLY/FOR/BDP versus GLY/IND; UMEC/VI/FF versus UMEC/VI; and GLY/FOR/B versus GLY/FOR), triple therapy resulted in RR of: 0.85 (95% CI: 0.72, 0.995; 15% difference; *p* = 0.043) [23]; 0.75 (95% CI: 0.70, 0.81; 25% difference; *p* < 0.001) [25]; and 0.48 (95% CI: 0.37, 0.64; 52% difference; *p* < 0.0001) [26]. The patient-based NNT with triple therapy compared with LAMA/LABA ranged between approximately 33 (IMPACT) and 50 (TRIBUTE). The event-based NNT ranged from 3 (IMPACT) to 11 (TRIBUTE).

In the studies that assessed single-inhaler triple therapy with a LABA/ICS (GLY/FOR/BDP versus FOR/BDP; UMEC/VI/FF versus VI/FF; UMEC/VI/FF versus FOR/B; GLY/FOR/B versus FOR/B), triple therapy resulted in RR of: 0.77 (95% CI: 0.65, 0.92; 23% difference; *p* = 0.005) [17]; 0.85 (95% CI: 0.80, 0.90; 15% difference; *p* < 0.001) [25]; 0.65 (95% CI: 0.49, 0.86; 35% difference; *p* = 0.002) [24] and 0.82 (95% CI: 0.58, 1.17; 18% difference; *p* = 0.2792) [26]. The patient-based NNT with triple therapy compared to LABA/ICS was around 25. The event-based NNT ranged from 6 (IMPACT) to 8 (TRILOGY). In the study that assessed single-inhaler triple therapy with a LAMA (GLY/FOR/BDP versus tiotropium [TIO]), triple therapy resulted in a RR of 0.80 (95% CI: 0.69, 0.92; 20% difference; *p* = 0.0025), with a patient-based NNT of around 25 and an event-based NNT of 9.

#### Prior exacerbation frequency subgroup

For the prior exacerbation frequency subgroup, triple therapy demonstrated a higher reduction in the rate of moderate and severe exacerbations in patients with a history of more exacerbations than those who experienced fewer exacerbations compared to LAMA/LABA (IMPACT [21]), LABA/ICS (TRILOGY) and LAMA (TRINITY). However, the converse was also found in comparisons with LAMA/LABA (TRIBUTE) and LABA/ICS (IMPACT [21]) where triple therapy demonstrated a higher reduction in the rate of moderate and severe exacerbations in patients who experienced fewer historical exacerbations compared to those with a higher frequency of historical exacerbations. A post-hoc analysis of data from the FULFIL study showed that, compared with LABA/ICS, triple therapy resulted in a significantly higher reduction in moderate and severe exacerbation rates in patients who had experienced ≥1 severe exacerbation, and 0/1 moderate exacerbations; patients who experienced ≥2 moderate exacerbations also experienced a rate reduction but the difference was not statistically significant [20]. There were no data relating to prior exacerbation frequency subgroups in the KRONOS study.

#### Eosinophil subgroup

For the eosinophil subgroup, in the TRINITY, TRIBUTE, IMPACT and KRONOS studies there was a higher reduction in the rate of moderate and severe exacerbations in patients with higher eosinophil counts than those with lower counts for triple therapy comparisons with LAMA/LABA (TRIBUTE, IMPACT and KRONOS) and LAMA (TRINITY). However, in the TRILOGY study (triple therapy [GLY/FOR/BDP] versus LABA/ICS), there was no association between blood eosinophil concentration and the rate of moderate and severe exacerbations. In the IMPACT study there was a higher reduction in the rate of moderate and severe exacerbations in patients with lower eosinophil counts than those with higher counts for triple therapy compared with LABA/ICS. In the KRONOS study (triple therapy [GLY/FOR/B] versus LABA/ICS), there were similar rates of moderate and severe exacerbations across most eosinophil subgroups. There were no data relating to eosinophil subgroups in the FULFIL study.

#### Smoking status subgroup

For the smoking status subgroup, there was a strong trend for a higher reduction in the rate of moderate and severe exacerbations in ex-smokers compared to smokers for triple therapy comparisons with LAMA/LABA in the largest study (IMPACT) and against LABA/ICS in TRILOGY. For the triple therapy comparisons with LAMA/LABA in TRIBUTE and LAMA in TRINITY, reduction in the rate of exacerbations was greater for smokers. There were no data relating to smoking status subgroups in the FULFIL or KRONOS studies.

### Secondary outcomes

#### Time to first exacerbation

**Fig. 4:**
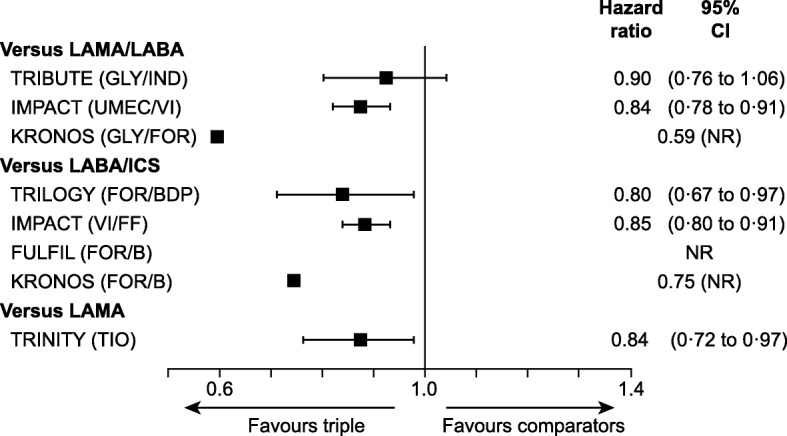
Time to first moderate or severe exacerbation. *CI* confidence interval, *COPD* chronic obstructive pulmonary disease, *FULFIL* Lung FUnction and quality of LiFe assessment in COPD with closed trIpLe therapy, *ICS* inhaled corticosteroids, *IMPACT* InforMing the PAthway of COPD Treatment, *LABA* long-acting β_2_ agonist, *LAMA* long-acting muscarinic antagonist, *NR* not reported, *TIO* tiotropium. Hazard ratios for time to first moderate or severe exacerbation with triple therapy compared with comparators

In the TRINITY, TRILOGY and IMPACT studies the time to first moderate or severe exacerbation was significantly extended with triple therapy compared with LAMA/LABA, LABA/ICS and LAMA (Fig. 4). In the KRONOS study, the time to first moderate or severe exacerbation was extended with triple therapy; the improvement was significant with LAMA/LABA (hazard ratio [HR]: 0.59; *p* < 0.0001 [Cox regression] and *p* = 0.0001 [log rank]), but not with LABA/ICS (HR: 0.75; *p* = 0.0635 [Cox regression] and *p* = 0.0281 [log rank]). The TRIBUTE study showed a similar time to first moderate or severe exacerbation with triple therapy and LAMA/LABA (HR: 0.90; 95% CI: 0.76, 1.06; *p* = 0.22). There were no data relating to time to first moderate or severe exacerbation in the FULFIL study.

#### Lung function

In the three studies that assessed the single-inhaler triple therapy GLY/FOR/BDP (TRINITY, TRILOGY and TRIBUTE), triple therapy resulted in a significant change from baseline in pre-dose FEV_1_ compared with LAMA (61 mL; 95% CI: 37, 86; *p* < 0.0001 [52-week data]) [16], LABA/ICS (81 mL; 95% CI: 52, 109; *p* < 0.001 [26-week data], 63 mL; 95% CI: 32, 94; *p* < 0.001 [52-week data]) [17] and LAMA/LABA (22 mL overall; *p* < 0.05) [23]. In terms of FEV_1_ change from baseline, patients were more likely to respond (defined as change from baseline in pre-dose FEV_1_ ≥ 100 mL) to triple therapy than to LAMA (odds ratio [OR]: 1.61; 95% CI: 1.34, 1.93; *p* < 0.0001 [26-week data], 1.62; 95% CI: 1.35, 1.95; *p* < 0.0001 [52-week data]) [16], LABA/ICS (OR: 2.30; 95% CI: 1.82, 2.91; *p* < 0.001 [26-week data], 2.06; 95% CI: 1.62, 2.62; *p* < 0.001 [52-week data]) [17] but not LAMA/LABA (OR: 1.19; 95% CI: 0.91, 1.55; *p* = 0.198]) [23]. A further post-hoc analysis of the TRINITY study considered different thresholds for FEV_1_ response. At week 26, the proportion of responders (using a 50 mL threshold definition) was 48.0% for single-inhaler GLY/FOR/BDP and 35.7% for TIO; using a 120 mL threshold the proportions were 36.7% for single-inhaler GLY/FOR/BDP and 25.3% for TIO [32].

In the two studies that assessed the single-inhaler triple therapy UMEC/VI/FF (FULFIL and IMPACT), triple therapy resulted in a significant change from baseline in pre-dose FEV_1_ compared with LABA/ICS (FOR/B, 171 mL; 95% CI: 148, 194; *p* < 0.001 [24-week data], 179 mL; 95% CI: 131, 226; *p* < 0.001 [52-week data] [24]; VI/FF, 97 mL; 95% CI: 85, 109; *p* < 0.001 [52-week data]) [25] and LAMA/LABA (54 mL; 95% CI: 39, 69; *p* < 0.001 [52-week data]) [25]. In terms of FEV_1_ change from baseline, patients were more likely to respond (defined as change from baseline in trough FEV_1_ ≥ 100 mL) to triple therapy than to LABA/ICS (FOR/B, OR: 4.03; 95% CI: 3.27, 4.97; *p* < 0.001 [24-week data], 4.79; 95% CI: 3.02, 7.61; *p* < 0.001 [52-week data]) [24]. FEV_1_ response was not reported in the IMPACT study.

In the study that assessed the single-inhaler triple therapy GLY/FOR/B (KRONOS), triple therapy resulted in a significant change from baseline in pre-dose FEV_1_ compared with LAMA/LABA (22 mL; 95% CI: 4, 39; *p* = 0.0139), LABA/ICS (74 mL; 95% CI: 52, 95; *p* < 0.0001) [26]. FEV_1_ response was not reported in the KRONOS study.

#### Quality of life

Across all the studies quality of life was assessed using the St. George’s Respiratory Questionnaire (SGRQ) [33]. In the three studies that assessed the single-inhaler triple therapy GLY/FOR/BDP (TRINITY, TRILOGY and TRIBUTE), triple therapy resulted in greater improvement in SGRQ total score compared with TIO (at all timepoints except week 26 [weeks 4 and 12: *p* < 0.001; week 40: *p* < 0.01; week 52: *p* < 0.05]) [16], LABA/ICS (at weeks 4, 12 and 52 [week 52 mean treatment difference: –1.69; 95% CI: − 3.20, − 0.17; *p* = 0.029]) [17] and LAMA/LABA (week 4 and 12: *p* ≤ 0.001; week 26: *p* < 0.05; week 40 and 52: *p* < 0.01) [23]. In terms of SGRQ response (defined as decrease from baseline in total score ≥ 4 units), triple therapy was superior to TIO (OR: 1.32; 95% CI: 1.10, 1.57; *p* = 0.0024 [26-week data], 1.33; 95% CI: 1.11, 1.59; *p* = 0.0019 [52-week data]) [16] and LABA/ICS (OR: 1.52; 95% CI: 1.21, 1.91; *p* < 0.001 [26-week data], 1.33; 95% CI: 1.06, 1.66; *p* = 0.014 [52-week data]) [17], but not LAMA/LABA (OR: 1.22; 95% CI: 0.99, 1.51; *p* = 0.068 [52-week data]) [23].

In the two studies that assessed the single-inhaler triple therapy UMEC/VI/FF (FULFIL and IMPACT), triple therapy resulted in a greater improvement in SGRQ total score compared with LABA/ICS (FOR/B, at week 24 [difference: − 2.2 units; 95% CI: − 3.5, − 1.0; *p* < 0.001] [24]; VI/FF, at week 52 [difference: –1.8; 95% CI: − 2.4, − 1.1; *p* < 0.001]) [25] and LAMA/LABA (at week 52 [difference: –1.8; 95% CI: − 2.6, − 1.0; *p* < 0.001]) [25]. In the IMPACT study, there were similar changes in SGRQ total score for LAMA/LABA and LABA/ICS [25]. In terms of SGRQ response (defined as decrease from baseline in total score ≥ 4 units), triple therapy was superior to LABA/ICS (FOR/B, OR: 1.41; 95% CI: 1.16, 1.70; *p* < 0.001 [24-week data] [24]; VI/FF, OR: 1.41; 95% CI: 1.29, 1.55; *p* < 0.001 [52-week data]) [25] and LAMA/LABA (OR: 1.41; 95% CI: 1.26, 1.57; *p* < 0.001 [52-week data]) [25].

In the study that assessed the single-inhaler triple therapy GLY/FOR/B (KRONOS), triple therapy resulted in nominally significant improvements in SGRQ total score over 24 weeks compared with LAMA/LABA (difference: –1.22; 95% CI: − 2.30, − 0.15; *p* = 0.0259), but not LAMA/ICS (difference: –0.45; 95% CI: − 1.78, 0.87; *p* = 0.5036) [26]. In terms of SGRQ response (defined as decrease from baseline in total score ≥ 4 units), triple therapy was associated with nominally significant improvements compared with LAMA/LABA (OR: 1.28; 95% CI: 1.01, 1.61; *p* = 0.0395) but not LABA/ICS (OR: 1.30; 95% CI: 0.97, 1.72; *p* = 0.0746) [26].

#### Safety

Across all the studies, SAEs were comparable between triple therapy and comparators. In the three studies that assessed the triple therapy GLY/FOR/BDP (TRINITY, TRILOGY and TRIBUTE), triple therapy was associated with a similar percentage of patients who experienced SAEs (13 [16]–15% [17, 23]) compared with TIO (15%) [16], LABA/ICS (18%) [17] and LAMA/LABA (17%) [23].

In the two studies that assessed the triple therapy UMEC/VI/FF (FULFIL and IMPACT), triple therapy was associated with a similar percentage of patients who experienced SAEs (5.4% [24] [24-week data], 10 [24]–22% [25] [52-week data]) compared with LABA/ICS (FOR/B, 5.7% [24-week data], 12.7% [52-week data] [24]; VI/FF, 21% [52-week data]) [25] and LAMA/LABA (23% [52-week data]) [25].

In the study that assessed the single-inhaler triple therapy GLY/FOR/B (KRONOS), triple therapy was associated with SAEs in 9% of patients compared with 11% for LAMA/LABA and 7% for LABA/ICS [26].

In all studies, except FULFIL and IMPACT, pneumonia was reported in 1 to 4% of patients, with similar incidences in the treatment groups within each study. Pneumonia was defined as an adverse event (AE) by the investigator in each of these studies and additionally, in the KRONOS study, was validated using supporting diagnostic and treatment criteria.

In the FULFIL study, there was a higher rate of pneumonia (defined as an AE with supporting radiography) for triple therapy compared with LABA/ICS up to 24 weeks (2.1% versus 0.8%); the rates were similar between the two groups at 52 weeks (1.9% versus 1.8%). In the IMPACT study there was a higher incidence of pneumonia (defined as an AE of special interest with supporting radiography) with the triple therapy (UMEC/VI/FF; 8%) and LABA/ICS (7%), than with LAMA/LABA (5%). The rate of pneumonia per 1000 patient-years was 95.8 for triple therapy, 61.2 for LAMA/LABA and 96.6 for LABA/ICS. For pneumonia defined as a SAE with supporting radiography, the rate per 1000 patient-years was 53.3 for triple therapy, 32.4 for LAMA/LABA and 47.7 for LABA/ICS. The risk of pneumonia was significantly higher with triple therapy than with LAMA/LABA, as assessed in a time-to-first-event analysis (HR: 1.53; 95% CI: 1.22, 1.92; *p* < 0.001) [25].

In the TRINITY, TRILOGY, TRIBUTE and FULFIL studies, mortality was reported to be similar in each arm and not related to study medication. A pooled analysis of the time to death in the TRINITY, TRILOGY and TRIBUTE studies did not show a significant reduction in the risk of developing a fatal event compared with ICS-free treatments (LAMA and LAMA/LABA, HR: 0.72; 95% CI: 0.49, 1.06; *p* = 0.096) [22]. In the IMPACT study, for the prespecified endpoint, there was a statistically significant reduction in the risk of time to on- and off-treatment all-cause mortality for triple therapy compared to LAMA/LABA (HR: 0.71; 95% CI: 0.51, 0.99; unadjusted *p* = 0.043) but not LABA/ICS. Similar results were seen when off-treatment information was included. The percentage of on- and off-treatment deaths in each arm were 2.14% with UMEC/VI/FF, 2.35% with VI/FF and 2.90% with UMEC/VI [25]. In the KRONOS study, there were two deaths in the LAMA/LABA group that were considered to be related to study treatment [26].

## Discussion

Overall, this systematic review demonstrates, that in patients with predominantly severe lung function impairment and a history of frequent exacerbations, single-inhaler triple therapy results in a reduction in moderate and severe exacerbations. Over 1 year, triple therapy reduced the frequency of moderate and severe exacerbations by 15 to 52% compared to LAMA/LABA, and 15 to 35% compared to LABA/ICS. The patient-based NNT ranged from around 25 to 50 (preventing one patient from having an event) and the event-based NNT ranged from around 3 to 11 (preventing one event). The absolute benefit appears to be greater in phenotypic subgroups with higher eosinophil counts or higher historical frequency of exacerbations and to some extent, ex-smokers. The reductions in exacerbations come at the expense of a significant increase in pneumonia for some regimens, including pneumonia as a SAE.

Single-inhaler triple therapy showed some improvements in lung function and health-related quality of life over comparators. However, for the responder analysis (where threshold definitions reflected minimal clinically-important differences), comparisons against LAMA/LABA were not significant, indicating the differences were small and potentially not clinically relevant.

Previous reviews assessing triple therapy, in general, have demonstrated a reduction in exacerbations and an improvement in lung function and health-related quality of life but with an increased risk of pneumonia [3–7]. Our findings are consistent with a previously published review of multiple and single inhaler triple therapies, which included three of the six single inhaler clinical trials included in the present study. Although results for single inhalers were not reported separately, the results indicate that the benefit of triple therapy in reducing the risk of exacerbation is greater in patients with higher eosinophil counts [7]. Overall, the magnitude of clinical benefit for single-inhaler triple therapy, particularly across multiple subgroups, has not been well documented, which was the focus of our study. This study is the first to systematically review and descriptively assess the relative and absolute efficacy and safety of all six single-inhaler triple therapy studies compared to dual and monotherapy and explore differences in efficacy between phenotypic subgroups.

We also critically assessed the evidence base for single-inhaler triple therapy, which is currently not well documented. There were important differences in study designs and populations that would give rise to significant heterogeneity if class-level or drug-level meta-analyses of single-inhaler triple therapies were conducted, meaning results of such studies would need to be treated with caution. First, prior triple therapy was allowed in some trials and not others. The UMEC/VI/FF studies (FULFIL and IMPACT) allowed patients on triple therapy at the time of randomisation to enter the trial. In the IMPACT study 35% of patients in both the LAMA/LABA and LABA/ICS arms experienced ICS withdrawal at the time of randomisation. Second, some trials allowed abrupt withdrawal of ICS prior to study start. In the IMPACT study, more than 70% of patients randomised to LAMA/LABA were receiving ICS at randomisation. In the TRIBUTE study, all patients were switched to LAMA/LABA at the start of the 2-week run-in period. This included 65% of patients who were taking LABA/ICS or LAMA/ICS for at least 2 months before study entry. No subgroup analyses were done according to previous treatment. Third, there were differences in the severity of COPD between the trials, particularly in relation to prior exacerbation history, with one study (KRONOS) including patients without a prior history of exacerbations, a group not recommended for treatment with triple therapy according to the recent GOLD update [2]. Fourth, different regimens were used, which may impact the risks and benefits observed. Finally, the IMPACT trial also allowed patients with a prior history of asthma. As these data were not recorded, however, it is difficult to know whether this influenced results. All of these factors could lead to exaggeration of the benefits of ICS. Some studies have shown that abrupt withdrawal of ICS may lead to an increase in exacerbations in some patients [34–36], unlike a managed stepwise withdrawal of ICS [37]. Class-level meta-analyses including free-combination triple therapies and limited data on single-inhaler triple therapies have found statistical and clinical heterogeneity to be a significant concern [6, 7].

The IMPACT study reported a benefit in the risk of time to all-cause mortality for triple therapy compared to LAMA/LABA. As none of the single-inhaler triple therapy studies were powered for a mortality outcome, these results should be treated with caution and investigated further. Two prospective, adequately-powered, large-scale studies with a primary outcome of mortality failed to demonstrate a significant reduction in mortality associated with the use of ICS-containing regimens [38, 39].

Our review attempted to determine whether the benefits associated with triple therapy in COPD were clinically meaningful by assessing and comparing relative and absolute improvements in outcomes as well as the NNT. These latter results are of interest as they translate the results of randomised trials into a value that clinicians can use in decision making. Focusing on the 12-month trials, we found relatively high NNTs for therapy at 12 months (> 20) in terms of reducing the numbers of individuals experiencing exacerbations but lower NNTs (3 to 11) for reducing one exacerbation per patient per year. The discrepancy between these two numbers suggests that the impact of triple therapy is not necessarily to increase the number of patients staying exacerbation free, but that the largest effect is in reducing the number of events in patients having multiple events. Such patients are most likely the high-risk individuals with a higher rate of baseline exacerbations and blood eosinophilia as suggested by the subgroup data. The evidence therefore supports a targeted approach to triple therapy use in those patients at highest risk of exacerbation with higher levels of blood eosinophilia. It also implies that the vast majority of patients with a lower baseline risk of exacerbation and lower blood eosinophilia may experience less benefit from ICS, as reflected in the GOLD update [2]. The increased risk of ICS, particularly in relation to pneumonia, is well documented [40], and likely to vary by treatment-related factors (e.g. regimen and dose) and study design-related factors (e.g. patient characteristics and definitions used) [41]. In the present study, the pneumonia risk for single-inhaler triple therapy ranged from no reported increase to a significant increase. Taking the study demonstrating the highest risk and translating it into an event-based number needed to harm (NNH) to induce pneumonia in at least one patient over one year, gives an NNH of 33.

The next step is to more clearly define patient groups where ICS are of benefit. This review demonstrated that patients with higher eosinophil counts, higher prior exacerbation frequency and ex-smokers, in general, benefited more from single-inhaler triple therapy; however, results for all three phenotypes were mixed. This may have resulted, in part, from the thresholds that were used in these studies. The GOLD 2019 document suggests that patients with blood eosinophil counts < 100 and ≥ 300 cells/μL point to subgroups with lower and higher likelihood of treatment benefit with ICS [2]. There is a need to validate and refine this threshold and identify other patient characteristics that can support the treatment decision to step up to triple therapy.

The limitations of our systematic review largely reflect the shortcomings of the included studies. First, there was limited information reported in the studies on subgroups and information needed to calculate patient-based NNTs therefore a number of the results were either calculated based on available data or estimated from forest plots or Kaplan–Meier curves. Second, interpretation of subgroup data needs to be treated with caution. Many subgroup analyses were not prespecified in the original study and studies were not powered to demonstrate an interaction effect. In addition, although the inclusion criteria for this review were carefully chosen to answer the research question, broadening the criteria, for example by including all types of randomised controlled trials of any duration, may have had an impact on the interpretation of the results. However, a review with broader inclusion criteria, presented similar overall findings [7].

Overall the evidence from these studies support the GOLD 2019 treatment strategy in that triple therapy should be reserved for a select group of patients with COPD with frequent exacerbations and higher eosinophil counts thereby maximising benefit and minimising risk [2]. Future research on specific patient phenotype thresholds that can support treatment and funding decisions is now required from well-designed, robust, clinical trials.

## Conclusions

Our analysis of single-inhaler triple therapy trials suggests that in the patient populations included in these studies, 25–50 patients would need to be treated with single-inhaler triple therapy to prevent one patient from having a moderate or severe exacerbation over one year compared with dual therapy, with an NNH for the pneumonia AE of around 33 and upwards. This emphasises that we must weigh benefits and risks of ICS in individual patients. Our analyses suggest benefit appears to be greatest in patients with higher eosinophil counts, greater historical frequency of exacerbations, and ex-smokers. This must be balanced against the increased risk for AEs including pneumonia.

## Supplementary information

**Additional file 1: Table S1.** Eligibility criteria. **Table S2.** Search strategy. **Table S3.** Overview of excluded studies during the full-text analysis stage in alphabetical order. **Figure S1.** Risk of bias assessment summary.

## Data Availability

All data generated or analysed during this study are included in this published article [and its supplementary information files].

## Abbreviations

AE: Adverse event
B: Budesonide
BDP: Beclomethasone
CAT: COPD assessment test
CI: Confidence interval
COPD: Chronic obstructive pulmonary disease
FEV_1_: Forced expiratory volume in one second
FF: Fluticasone furoate
FOR: Formoterol fumarate
FULFIL: Lung FUnction and quality of LiFe assessment in COPD with closed trIpLe therapy
GLY: Glycopyrronium bromide
GOLD: Global Initiative for Chronic Obstructive Lung Disease
HR: Hazard ratio
ICS: Inhaled corticosteroids
IMPACT: InforMing the PAthway of COPD Treatment
IND: Indacaterol
LABA: Long-acting β_2_ agonist
LAMA: Long-acting muscarinic antagonist
MF: Mometasone furoate
NNH: Number needed to harm
NNT: Number needed to treat
NR: Not reported
OR: Odds ratio
PRISMA: Preferred Reporting Items for Systematic Reviews and Meta-Analyses
RR: Rate ratio
SAE: Serious adverse event
SD: Standard deviation
SGRQ: St. George’s Respiratory Questionnaire
TIO: Tiotropium
UMEC: Umeclidinium
VI: Vilanterol
