# Supplementary material for: Single-inhaler triple therapy in patients with chronic obstructive pulmonary disease: a systematic review
Source: Respir Res. 2019 Nov 4;20:242. doi: 10.1186/s12931-019-1213-9 (PMC6829989; doi:10.1186/s12931-019-1213-9)
Supplement: Supplementary file 1 — Additional file 1: Table S1. Eligibility criteria. Table S2. Search strategy. Table S3. Overview of excluded studies during the full-text analysis stage in alphabetical order. Figure S1. Risk of bias assessment summary. [file 12931_2019_1213_MOESM1_ESM.pdf]

Additional file

Supplementary Table 1 Eligibility criteria

**Participants**	Adult patients with COPD
**Intervention**	Single-inhaler triple therapy<br>GLY/FOR/BDP, UMEC/VI/FF, GLY/FOR/B (PT010) and GLY/IND/MF (QVM149)
**Comparator**	LABA, LAMA, LAMA/LABA or LABA/ICS
**Outcomes**	Primary outcomes: the annual rate of moderate and severe exacerbations<br>Secondary outcomes: time to first exacerbation, lung function, quality of life and safety outcomes
**Study designs**	Parallel-group randomised controlled trials of ≥3 months duration

B budesonide, *BDP* beclomethasone, *COPD* chronic obstructive pulmonary disease, *FF* fluticasone furoate, *FOR* formoterol fumarate, *GLY* glycopyrronium bromide, *ICS* inhaled corticosteroids, *IND* indacaterol, *LABA* long-acting ß$_2$ agonist, *LAMA* long-acting muscarinic antagonist, *MF* mometasone furoate, *UMEC* umeclidinium, *VI* vilanterol

Supplementary Table 2 Search strategy

Database	Search terms
**Embase (OvidSP)**	P – Adult patients with COPD<br>chronic obstructive lung disease/ OR *obstructive airway disease/dt [Drug Therapy] OR ((obstruct* and (pulmonary or lung* or airway* or airflow* or bronch* or respirat*)) or COPD or emphysema or (chronic* and bronchiti*)).ti,ab,kw.<br><br>AND<br><br>I – Single-inhaler triple therapy<br>triple therap*.ti,ab,kw. OR beclometasone dipropionate plus formoterol fumarate plus glycopyrronium bromide/ OR beclometasone dipropionate plus formoterol fumarate/ and (glycopyrronium/ or glycopyrronium bromide/) OR (beclometasone/ or beclometasone dipropionate/) and (formoterol/ or formoterol fumarate/) and (glycopyrronium/ or glycopyrronium bromide/) OR (trimbow or (BDP adj3 FF adj3 GB) or (glycopyr* and formoterol and beclo*)).af. OR fluticasone furoate plus umeclidinium plus vilanterol/ OR umeclidinium plus vilanterol/ and (fluticasone/ or fluticasone furoate/) OR umeclidinium/ and (fluticasone/ or fluticasone furoate/) and vilanterol/ OR (trelegy or (FF adj3 UMEC adj3 VI) or ((umeclidinium or incruse) and vilanterol and (fluticason* or flovent or flixotide or flixonase or flonase))).af. OR formoterol fumarate plus glycopyrronium bromide/ and budesonide/ OR budesonide plus formoterol/ and (glycopyrronium/ or glycopyrronium bromide/) OR budesonide/ and (formoterol/ or formoterol fumarate/) and (glycopyrronium/ or glycopyrronium bromide/) OR (glycopyr* and formoterol and (budesonide or entocort or pulmicort)).af. OR indacaterol/ and mometasone furoate/ and (glycopyrronium/ or glycopyrronium bromide/) OR (glycopyr* and indacaterol and mometason*).af.<br><br>C – none

Database	Search terms
	O – none<br><br>AND<br><br>S – Randomised controlled trials<br>randomized controlled trial/ OR randomization.de. OR controlled clinical trial/ and (Disease Management or Drug Therapy or Prevention or Rehabilitation or Therapy).fs. OR *clinical trial/ OR placebo.de. (309585) OR placebo.ti,ab. OR trial.ti. (241328) OR (randomi#ed or randomi#ation or randomi#ing).ti,ab,kw. OR (RCT or "at random" or (random* adj3 (administ* or allocat* or assign* or class* or control* or determine* or divide* or division or distribut* or expose* or fashion or number* or place* or recruit* or subsitut* or treat*))).ti,ab,kw. OR ((singl$ or doubl$ or trebl$ or tripl$) adj3 (blind$ or mask$ or dummy)).mp. OR (control* and (trial or study or group) and (placebo or waitlist* or wait* list* or ((treatment or care) adj2 usual))).ti,ab,kw,hw.<br><br>Non-human research was excluded using filtering option
**MEDLINE (Ovid SP) includes Ovid MEDLINE(R) Epub Ahead of Print, In-Process & Other Non-Indexed Citations, Ovid MEDLINE(R) Daily and Ovid MEDLINE(R)**	P – Adult patients with COPD<br>Pulmonary Disease, Chronic Obstructive/ OR ((obstruct* and (pulmonary or lung* or airway* or airflow* or bronch* or respirat*)) or COPD or emphysema or (chronic* and bronchiti*)).ti,ab,kf.<br><br>AND<br><br>I – Single-inhaler triple therapy<br>triple therap*.ti,ab,kf. OR (trimbow or (BDP adj3 FF adj3 GB) or (glycopyr* and formoterol and beclo*)).af. OR (trelegy or (FF adj3 UMEC adj3 VI) or ((umeclidinium or incruse) and vilanterol and (fluticason* or flovent or flixotide or flixonase or flonase))).af. OR (glycopyr* and formoterol and (budesonide or entocort or pulmicort)).af. OR (glycopyr* and indacaterol and mometason*).af.<br><br>C – none<br><br>O – none<br><br>AND<br><br>S – Randomised controlled trials<br>controlled clinical trial.pt. OR randomized controlled trial.pt. OR (randomi#ed or randomi#ation or randomi#ing).ti,ab,kf. OR (RCT or "at random" or (random* adj3 (administ* or allocat* or assign* or class* or control* or crossover or cross-over or design* or determine* or divide* or division or distribut* or expose* or fashion or number* or place* or recruit* or subsitut* or treat*))).ti,ab,kf. OR placebo*.ab,ti,kf. OR (trial or study).ab,ti,kf. OR groups.ab. OR (control* and (trial or study or group*) and (placebo or waitlist* or wait* list* or ((treatment or care) adj2 usual))).ti,ab,kf,hw. OR ((single or double or triple or treble) adj2 (blind* or mask* or dummy)).ti,ab,kf. OR double-blind method/ or random allocation/ or single-blind method/

Database	Search terms
	Non-human research was excluded using filtering option
**The Cochrane Library, issue 3**	P – Adult patients with COPD<br>MeSH descriptor: [Pulmonary Disease, Chronic Obstructive] explode all trees OR ((obstruct* and (pulmonary or lung* or airway* or airflow* or bronch* or respirat*)) or COPD or emphysema or (chronic* and bronchiti*)):ti,ab,kw (Word variations have been searched)<br><br>AND<br><br>I – Single-inhaler triple therapy<br>"triple therap*":ti,ab,kw (Word variations have been searched) OR (trimbow or (BDP near/3 FF near/3 GB) or (glycopyr* and formoterol and beclo*)):ti,ab,kw (Word variations have been searched) OR (trelegy or (FF near/3 UMEC near/3 VI) or ((umeclidinium or incruse) and vilanterol and (fluticason* or flovent or flixotide or flixonase or flonase))):ti,ab,kw (Word variations have been searched) OR (glycopyr* and formoterol and (budesonide or entocort or pulmicort)):ti,ab,kw (Word variations have been searched) OR (glycopyr* and indacaterol and mometason*):ti,ab,kw (Word variations have been searched)<br><br>C – none<br><br>O – none<br><br>S – none
**Trial registries**	*ClinicalTrials.gov and WHO ICTRP*<br>COPD and triple therapy OR trimbow OR beclometasone and formoterol and glycopyrronium OR beclomethasone and formoterol and glycopyrronium OR trelegy OR fluticasone and umeclidinium and vilanterol OR formoterol and glycopyrronium and budesonide OR indacaterol and mometasone and glycopyrronium

C comparator, *COPD* chronic obstructive pulmonary disease, *I* intervention, *ICTRP* International Clinical Trials Registry Platform, *P* population, *O* outcome, *S* study type, *WHO* World Health Organization.

Supplementary Table 3 Overview of excluded studies during the full-text analysis stage in alphabetical order

Citation	Reason for exclusion
Boehringer Ingelheim. 2017. https://ClinicalTrials.gov/show/NCT03265145	No results available
Bremner PR, Birk R, Brealey N, Ismaila AS, Zhu CQ, Lipson DA. Single-inhaler fluticasone furoate/umeclidinium/vilanterol versus fluticasone furoate/vilanterol plus umeclidinium using two inhalers for chronic obstructive pulmonary disease: a randomized non-inferiority study. *Respir Res.* 2018;19(1):19	Wrong comparator (single-inhaler triple *versus* open triple)
Budenholzer B. Adding glycopyrronium to beclomethasone plus formoterol improved pulmonary function in COPD. *Ann Intern Med.* 2016;165(12):JC67	Publication type (comment, expert commentary, editorial)
Calverley PM. COPD therapy: if two is good, is three better? *Lancet.* 2016;388(10048):937–938	Publication type (comment, expert commentary, editorial)
Cazzola M, Rogliani P, Puxeddu E, Ora J, Matera MG. An overview of the current management of chronic obstructive pulmonary disease: can we go beyond the GOLD recommendations? *Expert Rev Respir Med.* 2018;12(1):43–54	Publication type (comment, expert commentary, editorial)
Chalmers JD. POINT: Should an attempt be made to withdraw inhaled corticosteroids in all patients with stable GOLD 3 (30% ≤ FEV(1) < 50% predicted) COPD? Yes. *Chest.* 2018;153(4):778–782	Publication type (comment, expert commentary, editorial)
Chatterjee A, Shah M, D'Souza AO, Bechtel B, Crater G, Dalal AA. Observational study on the impact of initiating tiotropium alone versus tiotropium with fluticasone propionate/salmeterol combination therapy on outcomes and costs in chronic obstructive pulmonary disease. *Respir Res.* 2012;13:15	Wrong intervention (open triple)
Chiesi Farmaceutici. 2011. https://clinicaltrials.gov/show/NCT01476813	Wrong intervention (open triple)
Chiesi Farmaceutici. 2013. https://clinicaltrials.gov/show/NCT01917331	No original data
Chiesi Farmaceutici. 2013. https://www.clinicaltrialsregister.eu/ctr-search/search?query=eudract_number:2013-000063-91	No original data
Chiesi Farmaceutici. 2014. https://ClinicalTrials.gov/show/NCT01911364	No original data
Chiesi Farmaceutici. 2014. https://www.clinicaltrialsregister.eu/ctr-search/search?query=eudract_number:2014-001487-35	Wrong comparator (single-inhaler triple *versus* open triple)
Chiesi Farmaceutici. 2014. https://www.clinicaltrialsregister.eu/ctr-search/search?query=eudract_number:2014-001704-22	No original data
Chiesi Farmaceutici. 2015. https://ClinicalTrials.gov/show/NCT02467452	Wrong comparator (single-inhaler triple *versus* open triple)
Chiesi Farmaceutici. 2015. https://ClinicalTrials.gov/show/NCT02579850	No original data
Chiesi Farmaceutici. 2016. https://ClinicalTrials.gov/show/NCT03197818	No results available
Edwards SJ, Gray J. PRS5 Budesonide/formoterol plus tiotropium (BUD/FORM + TIO) vs. salmeterol/fluticasone plus tiotropium (SALM/FLU + TIO): A systematic review and adjusted indirect comparison between two alternative triple treatments in chronic obstructive pulmonary disease (COPD). *Value Health.* 2010;13(7):A319	Wrong intervention (open triple)
Fabbri LM, Rabe KF, Martinez FJ, Ferguson GT, Wang C, Ichinose M, Bourne E, Ballal S, Darken P, Deangelis K, Aurivillius M, Dorinsky P, Reisner C. PA688 Impact of eosinophil levels on lung function and exacerbation benefits with co-suspension delivery technology budesonide/glycopyrronium/formoterol metered dose inhaler (BGF MDI) in COPD (KRONOS study subgroup analysis). Presented at: ERS International Congress; Paris, France; 15–19 September; 2018	No original data
Frith P, Ratnavadivel R, Thompson P, Bremner P, Chang CL, Day P, Frenzel C, Kurstjens N. Late-breaking abstract: Glycopyrronium once-daily significantly improves lung function and health status when added to fluticasone/salmeterol in patients with COPD: The GLISTEN study. *Eur Respir J.* 2014;44:P2811	Wrong intervention (open triple)
Frith P, Thompson P, Ratnavadivel R, Chang C, Bremner P, Day P, Frenzel C, Kurstjens N for the GLISTEN study group. Once-daily glycopyrronium significantly improves lung function and health status and is comparable to tiotropium when added to fluticasone/salmeterol in COPD patients: the GLISTEN study. *Respirology.* 2015;20 (Suppl 2):79	Wrong intervention (open triple)
Gaebel K, Blackhouse G, Robertson D, Xie F, Assasi N, McIvor A, Hernandez P, Goeree R. PRS7 Triple therapy for chronic obstructive pulmonary disease. *Value Health.* 2010;13(3):A197	Wrong intervention (open triple)

Citation	Reason for exclusion
GlaxoSmithKline 2014. http://www.ins.gob.pe/ensayosclinicos/rpec/recuperarECPBNuevoEN.asp?numec=042-14	No original data
GlaxoSmithKline. 2013. https://ClinicalTrials.gov/show/NCT01762800	Wrong intervention (open triple)
GlaxoSmithKline. 2013. https://ClinicalTrials.gov/show/NCT01957163	Wrong intervention (open triple)
GlaxoSmithKline. 2013. https://ClinicalTrials.gov/show/NCT02119286	Wrong intervention (open triple)
GlaxoSmithKline. 2013. https://www.clinicaltrialsregister.eu/ctr-search/search?query=eudract_number:2013-002239-44	Wrong intervention (open triple)
GlaxoSmithKline. 2014. https://ClinicalTrials.gov/show/NCT02164513	No original data
GlaxoSmithKline. 2014. https://www.clinicaltrialsregister.eu/ctr-search/search?query=eudract_number:2013-003073-10	No original data
GlaxoSmithKline. 2014. https://www.clinicaltrialsregister.eu/ctr-search/search?query=eudract_number:2013-003075-35	No results available
GlaxoSmithKline. 2015. https://ClinicalTrials.gov/show/NCT02345161	No original data
GlaxoSmithKline. 2015. https://www.clinicaltrialsregister.eu/ctr-search/search?query=eudract_number:2013-002238-19	Wrong intervention (open triple)
GlaxoSmithKline. 2016. http://www.ensaiosclinicos.gov.br/rg/RBR-4sxpff/	No original data
GlaxoSmithKline. 2016. https://ClinicalTrials.gov/show/NCT02729051	Wrong comparator (single-inhaler triple *versus* open triple)
GlaxoSmithKline. 2016. https://ClinicalTrials.gov/show/NCT02731846	No results available
GlaxoSmithKline. 2016. https://www.clinicaltrialsregister.eu/ctr-search/search?query=eudract_number:2015-005212-14	Wrong comparator (single-inhaler triple *versus* open triple)
GlaxoSmithKline. 2018. https://ClinicalTrials.gov/show/NCT03467425	No results available
GlaxoSmithKline. 2018. https://ClinicalTrials.gov/show/NCT03474081	No results available
GlaxoSmithKline. 2018. https://ClinicalTrials.gov/show/NCT03478683	No results available
GlaxoSmithKline. 2018. https://ClinicalTrials.gov/show/NCT03478696	No results available
Hanania NA, Crater GD, Morris AN, Emmett AH, O'Dell DM, Niewoehner DE. Benefits of adding fluticasone propionate/salmeterol to tiotropium in moderate to severe COPD. *Respir Med.* 2012;106(1):91–101	Wrong intervention (open triple)
Hanania NA, Niewoehner DE, Crater GD, Emmett A, Dell DO, Cicale MJ. Triple therapy of fluticasone propionate/salmeterol combination 250/50 mcg and tiotropium improves lung function compared to tiotropium monotherapy. *Chest.* 2010;138(4 Suppl):867A	Wrong intervention (open triple)
Herman JB, West FM, Zappetti D. Are We FULFIL-led by a Once-daily Triple-therapy Inhaler for Chronic Obstructive Pulmonary Disease? *Clin Pulm Med.* 2018;25(2):77–78	Publication type (comment, expert commentary, editorial)
Hinds DR, DiSantostefano RL, Le HV, Pascoe S. Identification of responders to inhaled corticosteroids in a chronic obstructive pulmonary disease population using cluster analysis. *BMJ Open.* 2016;6(6):e010099	Wrong intervention (no triple therapy)
Hoshino M, Ohtawa J. Effects of tiotropium and salmeterol/fluticasone propionate on airway wall thickness in chronic obstructive pulmonary disease. *Respiration.* 2013;86(4):280–287	Wrong intervention (open triple)
Hoshino M. Effects of combining tiotropium and salmeterol/fluticasone propionate on airway dimensions in patients with COPD. *Eur Resp J.* 2013;42:3033	Wrong intervention (open triple)
Ismaila AS, Birk R, Shah D, Zhang S, Brealey N, Risebrough NA, Tabberer M, Zhu CQ, Lipson DA. Once-daily triple therapy in patients with advanced COPD: healthcare resource utilization data and associated costs from the FULFIL trial. *Adv Ther.* 2017;34(9):2163–2172	Wrong outcomes
Johansson G, Janson C, Stallberg B, Lisspers K, Stratelis G, Jorgensen L., Larsson K. COPD exacerbations and pneumonia on triple therapy (fixed ICS/LABA combinations and tiotropium). Does the choice of ICS/LABA matter? *Am J Respir Crit Care Med.* 2013;187:A2442	Wrong intervention (open triple)
Kerwin E, Siler T, Tombs L, Sousa AR, Singletary K, Church A. Triple therapy of umeclidinium and inhaled corticosteroid/long-acting β_2-agonist (ICS/LABA) vs placebo + ICS/LABA in GOLD D patients. *Eur Respir J.* 2015;46:PA1490	Wrong intervention (open triple)

Citation	Reason for exclusion
Kraemer M, Ellis A, Baldwin M, Jansen JP, Capkun-Niggli G, Cope S. PRS5 Dual bronchodilation with indacaterol and tiotropium in combination versus triple therapy, fixed-dose combinations, and monotherapy in COPD – a network meta-analysis of FEV_1. *Value Health.* 2012;15(7):A560	Wrong intervention (open triple)
Kwak MS, Kim E, Jang EJ, Kim HJ, Lee CH. The efficacy and safety of triple inhaled treatment in patients with chronic obstructive pulmonary disease: a systematic review and meta-analysis using Bayesian methods. *Int J Chron Obstruct Pulmon Dis.* 2015;10:2365–2376	Wrong intervention (open triple)
Lee SD, Xie CM, Yunus F, Itoh Y, Ling X, Yu WC, Kiatboonsri S. Efficacy and tolerability of budesonide/formoterol added to tiotropium compared with tiotropium alone in patients with severe or very severe COPD: A randomized, multicentre study in East Asia. *Respirology.* 2016;21(1):119–127	Wrong intervention (open triple)
Lee SD, Xie C-M, Yunus F, Itoh Y, Su R. Efficacy and tolerability of budesonide/formoterol (B/F) added to tiotropium (T) vs T alone in East-Asian patients (pts) with severe/very severe chronic obstructive pulmonary disease (COPD). *Eur Respir J.* 2014:44:P282	Wrong intervention (open triple)
Lipari M, Wilhelm S, Kale-Pradhan P. Triple versus monotherapy in COPD: A meta-analysis. *Pharmacotherapy.* 2017;37(12): e236	No original data
Lipson DA, Barnacle H, Birk R, Brearley N, Zhu C-Q, Pascoe S. Improved lung function by 24-hour serial spirometric assessment with single inhaler triple therapy versus dual therapy in patients with advanced chronic obstructive pulmonary disease (COPD): Subgroup analysis of the global, randomized, phase III FULFIL st. *Am J Respir Crit Care Med*. 2017;195:A3605	Wrong outcomes
Lipson DA, Barnhart F, Brealey N, Day NC, Brooks J, Criner G, Dransfield MT, Halpin DMG, Han MK, Jones CE, Kilbride S, Lange P, Lomas DA, Martinez FJ, Singh D, Tabberer M, Wise RA, Pascoe SJ. Reduction in all-cause mortality with single inhaler triple therapy (FF/UMEC/VI) versus dual therapy (FF/VI and UMEC/VI) in symptomatic patients with COPD: prespecified analysis of the phase III IMPACT trial. *Am J Respir Crit Care Med* 2018; 197:A1015	No original data
Lipson DA, Barnhart F, Brealey N, Day NC, Brooks J, Criner G, Dransfield MT, Halpin DMG, Han MK, Jones CE, Kilbride S, Lange P, Lomas DA, Martinez FJ, Singh D, Tabberer M, Wise RA, Pascoe SJ. Single inhaler triple-therapy (ICS/LAMA/LABA) versus dual-therapy (ICS/LABA or LAMA/LABA) in patients with COPD at risk of exacerbations: efficacy and safety results of the phase III IMPACT trial. *Am J Respir Crit Care Med.* 2018; 197: A1014	No original data
Lipworth BJ, University of Dundee. 2006. https://ClinicalTrials.gov/show/NCT00996697	Wrong intervention (open triple)
Lomas D, Lipson D, Barnacle H, Birk R, Brealey N, Zhu CQ, Tabberer M. LATE-BREAKING ABSTRACT: Single inhaler triple therapy (ICS/LAMA/LABA) in patients with advanced COPD: Results of the FULFIL trial. *Eur Respir J.* 2016;48:PA4629	No original data
Malerba M, Nardin M, Santini G, Mores N, Radaeli A, Montuschi P. Single-inhaler triple therapy utilizing the once-daily combination of fluticasone furoate, umeclidinium and vilanterol in the management of COPD: the current evidence base and future prospects. *Ther Adv Respir Dis.* 2018;12:1753466618760779	No original data
Montuschi P, Malerba M, Macis G, Mores N, Santini G. Triple inhaled therapy for chronic obstructive pulmonary disease. *Drug Discov Today.* 2016;21(11):1820–1827	Wrong population
Naya IP, Lipson DA, Compton C. Prevention of early worsening of COPD with umeclidinium open triple therapy compared with inhaled corticosteroid/long-acting beta2-agonist alone: a pooled post hoc analysis. *Am J Respir Crit Care Med.* 2017;195:A3608	Wrong intervention (open triple)
Pascoe SJ, Lipson DA, Locantore N, Barnacle H, Brealey N, Mohindra R, Dransfield MT, Pavord I, Barnes N. A phase III randomised controlled trial of single-dose triple therapy in COPD: the IMPACT protocol. *Eur Respir J.* 2016;48(2):320–330	No results available
Pearl Therapeutics. 2015. https://ClinicalTrials.gov/show/NCT02465567	No results available
Pearl Therapeutics. 2015. https://ClinicalTrials.gov/show/NCT02497001	No results available
Pearl Therapeutics. 2015. https://ClinicalTrials.gov/show/NCT02536508	No results available
Pearl Therapeutics. 2016. https://ClinicalTrials.gov/show/NCT03262012	No results available
Rodrigo GJ, Plaza V, Castro-Rodríguez JA. Comparison of three combined pharmacological approaches with tiotropium monotherapy in stable moderate to severe COPD: a systematic review. *Pulm Pharmacol Ther.* 2012;25(1):40–47	Wrong intervention (open triple)

Citation	Reason for exclusion
Rojas-Reyes MX, García Morales OM, Dennis RJ, Karner C. Combination inhaled steroid and long-acting beta$_2$-agonist in addition to tiotropium versus tiotropium or combination alone for chronic obstructive pulmonary disease. *Cochrane Database Syst Rev.* 2016;(6):CD008532	Wrong intervention (open triple)
Sadigov A, Akhundov S, Bagirov R. Analysis of chronic obstructive pulmonary disease exacerbations with the triple therapy compared with dual and single bronchodilator therapy: which treatment is better for patients with severe disease? *Chest.* 2014;145(3 Suppl):425A	Wrong intervention (open triple)
Sadigov AS, Bagirov R, Abbasov C. Analysis of chronic obstructive pulmonary disease exacerbations with the triple therapy compared with dual treatment: is it better treatment tool for patients with severe disease? *Am J Respir Crit Care Med.* 2014;189:A3770	Wrong intervention (open triple)
Scuri M, Singh D, Fabbri LM, Guasconi A, Vezzoli S, Prunier H, Muraro A, Petruzzelli S, Papi A. Risk of pneumonia and exacerbations with single inhaler extrafine triple therapy compared to indacaterol/glycopyrronium: post-hoc analysis of the TRIBUTE study. *Am J Respir Crit Care Med.* 2018; 197: A3030	No original data
Scuri M, Singh D, Fabbri LM, Valente I, Guasconi A, Vezzoli S, Prunier H, Cohuet G, Muraro A, Petruzzelli S, Papi A. Single inhaler extrafine triple therapy vs indacaterol/glycopyrronium in COPD patients previously treated with LABA/LAMA: post-hoc analysis of the Tribute study. *Am J Respir Crit Care Med.* 2018; 197: A3043	Wrong population
Scuri M, Singh D, Papi A, Corradi M, Montagna I, Francisco C, Cohuet G, Vezzoli S, Muraro A, Petruzzelli S, Vestbo J. Cardiovascular safety of extrafine single inhaler triple combination of beclomethasone dipropionate, formoterol fumarate, and glycopyrronium bromide in COPD: results of safety analysis from the TRILOGY and TRINITY studies. *Thorax*. 2017;72(Suppl 3):A233	Wrong outcomes
Scuri M, Singh D, Papi A, Corradi M, Montagna I, Francisco C, Cohuet G, Vezzoli S, Muraro A, Petruzzelli S, Vestbo J. Cardiovascular safety of extrafine single inhaler triple combination of beclometasone dipropionate, formoterol fumarate, and glycopyrronium bromide in COPD: results of safety analysis from the TRILOGY and TRINITY studies. *Am J Respir Crit Care Med.* 2017; 195: A6448	Wrong outcomes
Scuri M, Singh D, Papi A, Corradi M, Montagna I, Francisco C, Cohuet G, Vezzoli S, Muraro A, Petruzzelli S, Vestbo J. Extrafine single inhaler triple therapy reduces use of rescue medication in COPD patients: Results from the TRINITY study. *Am J Respir Crit Care Med*. 2017;195:A6449	Wrong outcomes
Scuri M, Vestbo J, Papi A, Corradi M, Montagna I, Francisco C, Cohuet G, Vezzoli S, Muraro A, Petruzzelli S, Singh D. Effects of extrafine single inhaler triple therapy on lung function in COPD patients: results from responder analysis from the TRINITY study. *Am J Respir Crit Care Med*. 2017;195:A6450	Wrong intervention (open triple)
Siler T, Kerwin E, Sousa A, Donald A, Ali R, Church A. Efficacy and safety of once-daily umeclidinium added to fluticasone furoate/vilanterol in chronic obstructive pulmonary disease: Results of two replicate randomized 12-week studies. *Chest.* 2014;146(4 Suppl 2):340A	Wrong intervention (open triple)
Siler T, Kerwin E, Tombs L, Singletary K, Sousa AR, Church A. Triple therapy with umeclidinium plus fixed-combination inhaled corticosteroid/long-acting beta-agonist therapy in chronic obstructive pulmonary disease: Pooled results in placebo-controlled trials. *Am J Respir Crit Care Med.* 2015;191:A5778	Wrong intervention (open triple)
Siler TM, Kerwin E, Sousa AR Donald A, Ali R, Church A. Efficacy and safety of umeclidinium added to fluticasone furoate/vilanterol in chronic obstructive pulmonary disease: results of two randomized studies. *Respir Med.* 2015;109(9):1155–1163	Wrong intervention (open triple)
Singh D, Brooks J, Hagan G, Cahn A, O'Connor BJ. Superiority of "triple" therapy with salmeterol/fluticasone propionate and tiotropium bromide versus individual components in moderate to severe COPD. *Thorax.* 2008;63(7):592–598	Wrong intervention (open triple)
Singh D, Corradi M, Spinola M, Papi A, Usmani OS, Scuri M, Petruzzelli S, Vestbo J. Triple therapy in COPD: new evidence with the extrafine fixed combination of beclomethasone dipropionate, formoterol fumarate, and glycopyrronium bromide. *Int J Chron Obstruct Pulmon Dis.* 2017;12:2917–2928	No original data
Singh D, Vestbo J. Triple therapy in chronic obstructive pulmonary disease. *Am J Respir Crit Care Med.* 2017;196(8):1082–1083	Publication type (comment, expert commentary, editorial)

Citation	Reason for exclusion
Singh, D, Papi A, Corradi M, Montagna I, Francisco C, Cohuet G, Vezzoli S, Muraro A, Petruzzelli S, Scuri M, Vestbo J. LATE-BREAKING ABSTRACT: TRILOGY: a phase III study to evaluate the efficacy and safety of an extrafine triple combination of beclometasone dipropionate (BDP), formoterol fumarate (FF), and glycopyrronium bromide (GB) pMDI (CHF5993) in COPD patients. *Eur Respir J.* 2016;48:PA995	No original data
Sousa AR, Riley JH, Church A, Zhu CQ, Punekar YS, Fahy WA. The effect of umeclidinium added to inhaled corticosteroid/long-acting β2-agonist in patients with symptomatic COPD: a randomised, double-blind, parallel-group study. *NPJ Prim Care Respir Med.* 2016;26:16031	Wrong intervention (open triple)
Tabberer M, Lomas D, Barnacle H, Birk R, Brealey N, Zhu C-Q, Pascoe S, Locantore N, Lipson D. Single inhaler triple therapy (ICS/LAMA/LABA) in patients with advanced COPD: patient reported symptom and inhaler preference results from the FULFIL trial. *Am J Respir Crit Care Med*. 2017;195:A5484	No original data
Thompson P, Frith P, Frenzel C, Kurstjens N for the GLISTEN study group. Randomized controlled trial of glycopyrronium added to fixed combination salmeterol-fluticasone in COPD: Primary care and specialist site differences in the GLISTEN study. *Respirology.* 2015;20(Suppl 2):80	Wrong intervention (open triple)
Triple therapy for moderate-to-severe COPD: Marginal benefits. *Drug Ther Bull.* 2016:54(12):135–136	Publication type (comment, expert commentary, editorial)
Vestbo J, Corradi M, Montagna I, Cohuet G, Francisco C, Vezzoli S, Muraro A, Petruzzelli S, Scuri, M, Singh D. LATE-BREAKING ABSTRACT: TRINITY: A phase III study to compare the efficacy and safety of an extrafine triple combination of beclometasone dipropionate (BDP), formoterol fumarate (FF), and glycopyrronium bromide (GB) pMDI (CHF5993) with tiotropium (Tio) and a free triple combination of BDP/FF (Foster®) + Tio in COPD patients. *Eur Respir J.* 2016;48;OA1972	No original data
Wheeler K. Umeclidinium triple therapy for patients with COPD: Two studies. *Drug Topics.* 2016;160(5)	Wrong intervention (open triple)
Zhu Y, Zhang T, Li H, Yang Y, Chen Q, Kong L, Tai B. Discovering the relative efficacy of inhaled medications for chronic obstructive pulmonary disease: multiple treatment comparisons. *Cell Physiol Biochem.* 2017;41(4):1532–1546	Wrong intervention (open triple)

Supplementary Figure 1 Risk of bias assessment summary

	Selection bias (random sequence generation)	Selection bias (allocation concealment)	Performance bias (blinding of participants & personnel)	Detection bias (blinding of outcome assessment)	Attrition bias (incomplete outcome data)	Reporting bias (selective reporting)	Other bias
**TRINITY**	Green	Green	Green	Green	Green	Green	Green
**TRILOGY**	Green	Green	Green	Green	Green	Green	Green
**TRIBUTE**	Green	Green	Green	Green	Green	Green	Green
**FULFIL**	Green	Yellow	Yellow	Yellow	Green	Green	Green
**IMPACT**	Green	Green	Green	Yellow	Green	Green	Green
**KRONOS**	Green	Green	Green	Green	Green	Green	Green

COPD chronic obstructive pulmonary disease, *FULFIL* Lung FUnction and quality of LiFe assessment in COPD with closed trIpLe therapy, *IMPACT* InforMing the PAthway of COPD Treatment. Green: low risk of bias; yellow: unclear risk of bias.
